# Neuro-Signals from Gut Microbiota: Perspectives for Brain Glioma

**DOI:** 10.3390/cancers13112810

**Published:** 2021-06-04

**Authors:** Giuseppina D’Alessandro, Clotilde Lauro, Deborah Quaglio, Francesca Ghirga, Bruno Botta, Flavia Trettel, Cristina Limatola

**Affiliations:** 1 Department of Physiology and Pharmacology, Sapienza University, 00185 Rome, Italy; giuseppina.dalessandro@uniroma1.it (G.D.); clotilde.lauro@uniroma1.it (C.L.); flavia.trettel@uniroma1.it (F.T.); 2 IRCCS Neuromed, 86077 Pozzilli, IS, Italy; 3 Department of Chemistry and Technology of Drugs, “Department of Excellence 2018−2022”, Sapienza University, P.le Aldo Moro 5, 00185 Rome, Italy; deborah.quaglio@uniroma1.it (D.Q.); francesca.ghirga@uniroma1.it (F.G.); bruno.botta@uniroma1.it (B.B.); 4 Department of Physiology and Pharmacology, Sapienza University, Laboratory Affiliated to Istituto Pasteur Italia, 00185 Rome, Italy

**Keywords:** glioma, microbiota, gut-brain axis, neurotransmitters, cell proliferation

## Abstract

**Simple Summary:**

In the last few years, a lot of evidence demonstrated an unexpected bidirectional communication among the gut microbes and the brain. Gut microbiota derived molecules may affect the nervous system in physiological and pathological conditions, even modulating neurotransmitter levels. Here, we summarize the effects of neurotransmitters on the proliferation and differentiation of neuronal precursor cells in the adult brain, and in brain gliomas. Further, we discuss the hypothesis that modulation of neurotransmitters by gut microbiota might impact the development and progress of brain tumor, specifically glioma. Further investigation on the mechanisms involved in the bidirectional gut-brain communication is required to identify new molecular and cellular targets involved in the dysregulation of brain homeostasis occurring in glioma.

**Abstract:**

Glioblastoma (GBM) is the most aggressive form of glioma tumor in adult brain. Among the numerous factors responsible for GBM cell proliferation and invasion, neurotransmitters such as dopamine, serotonin and glutamate can play key roles. Studies performed in mice housed in germ-free (GF) conditions demonstrated the relevance of the gut-brain axis in a number of physiological and pathological conditions. The gut–brain communication is made possible by vagal/nervous and blood/lymphatic routes and pave the way for reciprocal modulation of functions. The gut microbiota produces and consumes a wide range of molecules, including neurotransmitters (dopamine, norepinephrine, serotonin, gamma-aminobutyric acid [GABA], and glutamate) that reach their cellular targets through the bloodstream. Growing evidence in animals suggests that modulation of these neurotransmitters by the microbiota impacts host neurophysiology and behavior, and affects neural cell progenitors and glial cells, along with having effects on tumor cell growth. In this review we propose a new perspective connecting neurotransmitter modulation by gut microbiota to glioma progression.

## 1. Introduction

Glioma is the most common primary malignant tumor of the central nervous system (CNS) in adults, with 50% of patients showing the most aggressive form, glioblastoma (GBM) [1]. GBM is characterized by high cell proliferation, active angiogenesis, and invasion capability. Despite the multimodal therapy approach (surgery, chemotherapy, and radiotherapy) the median survival time is only 14–15 months [2]. It is therefore urgent to develop further therapeutic strategies, expanding the research field to new tumor cell-host interactions to identify druggable cellular and molecular pathways. Several emerging pieces of evidence show a strong connection between microbiota and CNS functions, in physiological and pathological conditions. It is well accepted that the composition of gut microbiota can influence mood, behavior and cognition and that it synthesizes, modulates and responds to several neurotransmitters (e.g., dopamine, serotonin [5-hydroxytryptamine or 5-HT], norepinephrine and gamma-aminobutyric acid [GABA]) [3,4], which are deeply involved in those functions. Thus, since a functional axis exists between gut microbiota and brain, deeper investigations are needed to elucidate the underlying mechanisms, particularly in the context of brain tumors. It is widely accepted that neurotransmitters can be produced by microbes, which in turn can be modulated by neurotransmitters with effects on normal brain functions [5]. However, no investigations have been made in the context of glioma. Neurotransmitters act at synaptic level as chemical messengers allowing communication throughout the nervous system and with its effectors. In addition, they play extra-synaptic roles mediating the proliferation and differentiation of neuronal progenitors in the stem cell niches of the brain. In this contest, neurotransmitters act as positive or negative inducers of neural cell proliferation, and similar activity have been reported on glioma cells.

In this review, we report the evidence that gut microbiota produces, releases, and modulates neurotransmitter levels, and that these molecules play a role in regulation of the proliferation of neural progenitor and glioma cells. Overall, we highlight the need of further investigations on the interaction between gut-brain axis and glioma, in order to identify new targetable pathways as novel co-adjuvants of anti-glioma therapies.

## 2. Gut Microbiota Influences the CNS: The Gut-Brain Axis

In the gastrointestinal (GI) system resides the most abundant microbial population of the human body; the majority of these microorganisms include *bacteria*, *archaea*, *fungi*, *protozoa* and *viruses*, and are also referred as “gut microbiota” [6,7,8,9]. During the past decades, accumulating evidence shows that gut microbiota is essential not only to ensure the metabolic and immune functions [10], but also for neurogenesis [11] and brain development [12]. Gut microbiota interacts with the enteric and CNS through complex and in part unknown bidirectional signaling along the gut-brain-axis [13]. Host communicates with gut microbes releasing molecules that are recognized by commensal bacteria which, in turn, release bioactive molecules via humoral, endocrine, immune and neuronal pathways affecting gut and brain functions. This bidirectional communication allows CNS modulation of gut functions, such as motility, secretion, and immune function by the CNS; conversely, sensory visceral signals from the gut may influence brain activity and mood states [14,15]. Microbiota has been shown to influence the homeostasis of CNS through the immune, circulatory, and neural pathways [13]. It was demonstrated that there is an interplay between microbiota and immune system, and that metabolites produced from intestinal microbiota are able to activate the immune cells. For example, short-chain fatty acids (SCFAs), on the one hand, can activate inflammasome also through G-protein coupled receptor dependent mechanism [16]; on the other hand, they exhibit a protective effect in inflammatory reactions [17]. In the brain of antibiotic-treated mice it was observed an inhibition of brain-

Derived neurotrophic factor (BDNF) expression, and the activation of NF-kB in the hippocampus, resulting in neuroinflammation and anxiety-like behavior [18]. Since gut microbiota modulates CNS through different pathways including the immune system, it is reasonable to consider its impact on brain disorders where inflammation plays a role.

### 2.1. Gut Microbiota and Brain Disorders

One first scientific evidence obtained in Rhesus monkey showed a link between the gut microbiome and stress-related behaviors. Early-life stress modifies gut microbiota composition in Rhesus monkeys, and these changes correlate not only with anxiety-related behaviors, but also with elevated levels of stress hormones in the serum [19].

Research then mainly focused on mouse models where the gut bacteria were eliminated using broad-spectrum antibiotics or germ-free (GF) mouse lines. These bacteria-depleted animals exhibit significant alterations in neurophysiology and behavior compared to normal mice, suggesting a role of gut microbiota in modulation of CNS functions [20]. GF mice show a higher production of stress hormones and lower level of BDNF in the hippocampus, which were reversed by gut colonization with selected bacteria, suggesting a link among gut microbiota composition and stress responses [21]. Thereafter, several investigators extended these studies using different animal models, including antibiotic-treated rats and zebrafish, confirming that changes in gut microbiome alter stress-related behaviors across different organisms [12,22,23,24]. In human, evidence of a relation between GI pathology and neuropsychiatric disorders has been shown in anxiety, depression, and autism [25]. Moreover, a correlation with gut dysbiosis has been identified in preclinical models of Autism Spectrum Disorder (ASD) [26], Parkinson’s Disease (PD) [27], Alzheimer’s Disease (AD) [28], Multiple Sclerosis (MS) [29,30] and ischemic stroke [31].

In a Maternal Immune Activation (MIA) mouse model of autism, it has been found that GI altered permeability and behavioral abnormalities in offspring mice were corrected upon oral administration of a human commensal bacterium, *Bacteroides fragilis*. These findings highlight a gut-microbiome-brain axis in ASD and identify a potential probiotic therapy for GI and behavioral symptoms of autism [26].

PD is characterized by the loss of dopaminergic neurons in the substantia nigra, together with the accumulation of α-synuclein and Lewy bodies in neurons [32]. Sampson and collaborators demonstrated that chronic antibiotics treatment of α-synuclein overexpressing (ASO) adult mice induced less severe signs of pathophysiology. Moreover, depletion of gut microbes in young ASO mice inhibited the later disease progression [28], suggesting a role of microbiota in the evolution of PD.

AD is depicted by the progressive loss of neurons and synaptic functions, together with extracellular deposition of amyloid-β (Aβ) peptide and hyper-phosphorylated protein tau in neurons [33]. It was shown that Aβ mutant GF mice have a reduced cerebral pathology compared to control mice [34]. In line with this finding, AD mice treated with antibiotics had reduced Aβ deposition and milder neuropathological phenotype [29]. These observations indicate that microbiota might contribute to AD pathogenesis.

Experimental Autoimmune Encephalomyelitis (EAE) is a widely used mouse model of MS [35]. It was shown that the development of EAE symptoms was reduced in GF mice [36]. Moreover, MS patients have increased levels of specific gut microbes compared to healthy controls, and fecal microbiota transplantation from MS patients to EAE-GF mice induced a worsening of the disease [31]. Interestingly MS patient treatment with multi-strain probiotic leads to enrichment of specific microbial species in the gut, together with the inhibition of inflammation [37].

In the Medial Cerebral Artery Occlusion (MCAO) mouse model of ischemic stroke, antibiotics treatment reduced the ischemic brain injury, and this effect was transmissible by fecal transplants [31]. In addition, the authors demonstrated that alteration of intestinal flora affects immune cells, reducing neuroinflammation [31].

It has been shown that the crosstalk between gut microbiota and the brain might have a crucial impact also in brain tumors [38,39] even if more preclinical and clinical research are needed to define the molecular and cellular mechanisms involved. In this context it is therefore interesting to analyze which are the molecules that regulate the bidirectional communication between gut and brain.

### 2.2. Neuro-Signals from Gut Microbiota

**Figure 1:**
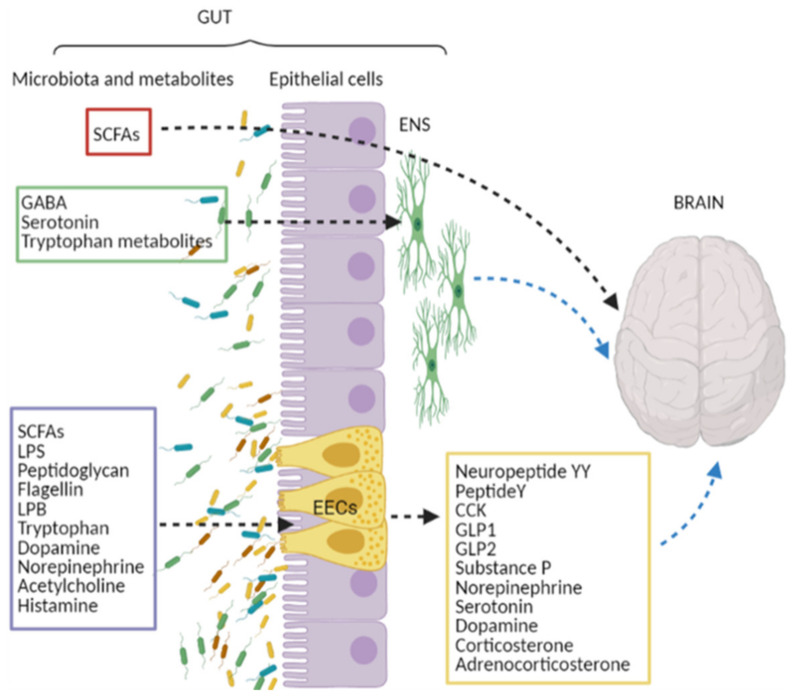
Neuro-signals from the gut. Bacterial derived molecules reported in (i) violet square can stimulate enteroendocrine cells (EECs) to release neuroactive molecules (yellow square), which affect brain functions through the vagal route (dotted blue arrow); (ii) green square can act on neurons of enteric nervous system (ENS) modulating brain functions through the vagal nerve; (iii) red square directly affect brain function passing the blood-brain barrier. SCFAs, short chain fatty acids; GABA, g-amino butyric acid; LPS, lipopolysaccharide; LBP, LPS binding protein; CCK, cholecystokinin; GLP-1/GLP2, glucagon-like peptide 1 and 2.

Different cellular and molecular pathways act along the gut-microbiota axis, and although an important role is played by gut hormones secreted by the enteroendocrine cells (EECs), strong attention is paid to neuroactive molecules produced by gut bacteria, as seen in Figure 1.

The CNS receives signals from the gut, which in turn influences CNS functions, primarily through the immune system, the release of neurotransmitters, and the involvement of the autonomic nervous system (ANS) with the vagal nerve, the enteric nervous system (ENS), using enteroendocrine signaling and metabolites produced by microbes [40].

Certainly, molecules that play an important role in the gut-brain crosstalk are the hormones secreted by the EECs whose release is also influenced by the diversity and composition of gut bacteria [41]. It has been shown that bacterial metabolites, such as lipopolysaccharide (LPS), SCFAs, and tryptophan, stimulate the EECs of the gut epithelium to produce neuropeptides, including peptide YY, neuropeptide Y, cholecystokinin, glucagon-like peptide (GLP)-1 and 2, and substance P. These neuropeptides cross the lamina propria, move along the bloodstream and reach local receptors, affecting ENS neurons and/or extrinsic vagal innervation [42,43]. The gut microbiota may also modulate the production of neurotransmitters and neuromodulators. It has been reported that the levels of norepinephrine, serotonin, and dopamine were decreased in GF mice, while those of GLP-1, corticosterone, and adrenocorticosterone were increased [44], suggesting that gut microbiota is able to promote the release of gut hormones from the EECs through metabolites or bacterial components [45,46,47,48].

However, bacteria have the potential to produce a range of major neurotransmitters such as dopamine, noradrenaline, serotonin, GABA, acetylcholine and histamine [49,50,51,52,53,54,55,56,57,58,59,60,61,62], and tryptophan metabolites [63,64] in addition to products suchLPS, LPS binding protein (LBP), peptidoglycan, and flagellin, which sustain the gut-brain axis communication [65].

Among the neuroactive substances and metabolites derived from gut microbiota, serotonin, GABA, and tryptophan metabolites, are not able to influence the CNS directly since they do not pass the blood–brain barrier (BBB). However, these molecules might exert their effects crossing the gut mucosal layer and interacting with cells in the enteric nervous system permitting signals to reach the nervous system directly [66]. Concerning the neuroactive SCFAs, it has been suggested that in addition to inducing neuropeptide production by EECs, they might also reach the brain crossing the BBB [67], possibly through the abundantly expressed monocarboxylate transporters (MCTs) on endothelial cells [68].

In summary, it has become clear that gut bacteria are able to influence the nervous system with different mechanisms including the stress-associated hypothalamic-pituitary-adrenal (HPA) axis modulation [21], vagal nerve stimulation [69,70], and SCFAs secretion, wich can reach the brain and activate microglial cells [71]. Moreover, gut bacteria can affect permeability of BBB [72] and modulate neurotransmitters through host biosynthesis pathways.

### 2.3. Modulation of Neurotransmitter Levels by Gut Microbiota

In addition to producing a number of neurotransmitters [73], gut microbes also modulate the levels of host neurotransmitters such as histamine [74], nitric oxide (NO) [75], neuropeptides [46], dopamine, norepinephrine, and serotonin, among others [44]. Studies on GF mice show that the absence of microbial colonization modifies the neurotransmitter turnover in the host CNS and ENS [76]. It was shown that a mixture of 46 *Clostridium* species could restore dopamine and norepinephrine levels in the cecal lumen of GF mice [73], but it remains to be understood whether this effect is due to a direct production of neurotransmitter or to the modulation of host production. GF mice also display an increased turnover rate of dopamine and norepinephrine in the brain [12], which could reduce the pools in systemic circulation independent of microbial production. It has been shown that the oral supplementation of *Enterococcus faecium* and *Lactobacillus rhamnosus* to young mice increased brain dopamine level [77]. Moreover, the gut microbiota can produce specific cofactors such as tetrahydrobiopterin (BH4), fostering tyrosine hydroxylase (TH) activity in the brain, with consequent increase in the level of dopamine [78].

GF mice also display an increased turnover rate of serotonin in the brain [12] and a significant reduction of serotonin in the blood and colon in comparison with control mice [67]. Administration of *Lactobacillus plantarum* to GF mice also significantly increased serotonin and dopamine levels in the striatal brain region [79]. In the brain, the influence of microbiota on serotonin is controversial: in GF mice the serotonin turnover increased in the striatum, but its levels were not changed [12]; on the other hand, the hippocampal regions of GF mice had increased levels of both serotonin and 5-hydroxyindoleacetic acid (5-HIAA), which is the main catabolic product of serotonin [80].

The gut microbiota also affects the level of circulating GABA, as shown in GF animals where GABA levels are reduced in gut lumen, in serum, but not in the brain [81]. It was demonstrated that *Lactobacillus rhamnosus* increases brain GABA level [82], and its administration reduces depressive- and anxiety-like behaviors, together with modifications in the expression of mRNA for cerebral GABA receptors [70]. Recently, it has been shown that *L. rhamnosus* JB-1, administration to mice induced a long-lasting enhancement of GABA as well as glutamate/glutamine brain levels, suggesting that the gut microbiota may regulate the biosynthetic pathways involved in glutamate production directly in the brain since amino acids do not cross the BBB under physiological conditions [82,83].

Moreover, the gut microbiota may indirectly influence the glutamatergic pathway controlling L-tryptophan metabolism; in fact, L-tryptophan contributes to the synthesis of serotonin, Kynurine (Kyn), and indole derivates in the gut [84]. Two downstream products of Kyn are KynA and quinolic acid. The first is an antagonist at the glycine site of N-methyl-D-aspartate (NMDA) receptors and is able to reduce excitotoxic damage in the nervous system; the second is an agonist of NMDA receptor and has neurotoxic and proinflammatory effects [85,86].

In this view, cerebral neurotransmitter modulation (see Table 1) might have an important role in the control of gut-brain axis signaling, especially because an imbalance of neurotransmission represents a key pathophysiological factor contributing to the development of several CNS disorders [87].

## 3. Neurotransmitters beyond the Neuronal Function

It is well established that neurotransmitters are key factors responsible for neuronal communication acting as synaptic chemical messengers that mediate transmission of information throughout the entire nervous system [88]. However, the presence of neurotransmitters already in the embryonal brain, long before the generation of synapses, suggests a role for such molecules beyond synaptic neurotransmission [89,90]. Indeed, pleiotropic functions of neurotransmitter as modulators of CNS development [91,92,93] have been documented in the last two decades. In different cerebral regions, neurotransmitters act locally or at distal loci as regulators of cell proliferation [94,95], neurogenesis [96,97], neuronal migration [98,99,100], synaptic maturation [101,102,103], neurite growth [104], circuit maturation [105] and programmed cell death [106,107,108]. The regulation of cell proliferation during brain development includes the ability of neurotransmitters to regulate DNA synthesis. GABA and glutamate, acting on GABA-A and alpha-amino-3-hydroxy-5-methyl-4-isoxazole propionic acid (AMPA) receptors, regulate the timing and terminate neuronal differentiation during corticogenesis inhibiting DNA synthesis and blocking transition to S phase [109]. Conversely, glutamate and NMDA receptors increase DNA synthesis and precursor cells’ proliferation in the striatum [110]. Moreover, several recent studies strongly suggest that neurotransmitters could act as growth regulators or morphogen-like signaling molecules able to regulate neuronal precursor cell (NPC) proliferation during cortical development [111,112]. However, neurotransmitters are able to modulate cell proliferation also in the adult brain, both in physiological condition and pathological conditions such as tumor brain, as follows.

### 3.1. Neurotransmitters Influence Physiological Cell Proliferation in Adult Brain

Beyond regulating cell proliferation during brain development, neurotransmitters as dopamine, norepinephrine, serotonin, GABA [113], and glutamate [114] also modulate cell proliferation of progenitor cells in adult brain (see Table 2).

#### 3.1.1. Dopamine

Dopamine is a monoamine that acts on metabotropic D1-like and D2-like receptor families, coupled to Gs alpha subunit or Gi alpha subunits, respectively.

In the adult brain, the neuronal stem cells in the subventricular zone (SVZ) respond to dopamine increasing cell proliferation [115]. Specifically, it has been shown that ablation of dopaminergic neurons reduces the proliferation of progenitors in the SVZ [116]; this reduction can be counteracted by dopamine agonists, a treatment sufficient to increase progenitor cell proliferation [117]. However, other studies have shown that the blockade of dopamine receptors increases proliferation [118,119]: these contrasting results might be explained by the activity of different dopamine receptors expressed on the same cells.

In addition, dopamine has been shown to regulate cell-cycle status of neuronal progenitor cells depending on the engagements of the specific dopamine receptor subtype. D1-like receptors block the entry of cells in S-phase, while D2-like receptors promote entry [120,121]. Further, the dopamine acting on D3 receptor blocks the maturation of oligodendrocyte progenitor cells (OPC) into mature cells [122].

#### 3.1.2. Serotonin

The role of serotonin in regulating cell proliferation in adult mature brain has also been documented. Depletion of serotonergic neurons, as well as the blockade of 5-HT1A serotonergic receptor, decreased proliferation in the dentate gyrus (DG) and SVZ [123,124], while elevating the level of serotonin by pharmacological or genetic manipulation, increased DG proliferation [125,126]. Similar to the other monoamine, serotonin influences OPC differentiation, maintaining OPC population and blocking their maturation [127]. In addition to regulate cell proliferation, serotonin exerts trophic roles [128,129] and mediates neurotrophic changes through 5-HT1A receptors, similar to those induced by peptide growth factors [130,131]. These findings confirm a role for serotonin in shaping adult brain.

#### 3.1.3. Norepinephrine

Norepinephrine depletion decreases the proliferation but not the differentiation of granule progenitor cells in adult hippocampus [132].

In line with this result, it has been shown that, in the sub-granular zone of the hippocampus of adult mice, norepinephrine directly activates self-renewing and multipotent neural precursor cells with a mechanism that requires the activity of β3 adrenergic receptor (β3 AR) [133]. In vivo, the intra-hippocampal injection of selective β3 AR agonist and treatment with antidepressants that block the reuptake of norepinephrine determine an increase of hippocampal precursor proliferation [133]. In particular, it has been shown that it is the balance between α2- and β-adrenergic receptor activity that regulates precursor cell activity and hippocampal neurogenesis [134]. In contrast, in the SVZ of the lateral ventricles, norepinephrine acts as a negative regulator of neuronal stem cell proliferation [135]. Thus, norepinephrine exerts opposite effects in the two main stem cell niches in the adult brain.

#### 3.1.4. GABA

A key role for GABA has been evidenced as regulator of cell proliferation in the SVZ of adult brain; in particular, a non-synaptic GABA signaling has been identified between neuroblasts and glial fibrillary acidic protein (GFAP)-stem cells that represent a feedback signal from neuroblast to limit the cell cycle progression of GFAP-stem cell [136]. Moreover, extra-synaptic GABA, together with glutamate, play an essential role in tuning neuroblast formation and migration in post-natal forebrain [137,138,139]. In another neurogenic niche of the adult brain, the sub-granular zone of the hippocampal DG, non-synaptic tonic GABA release from interneurons maintains the quiescence of neuronal precursor cells (NPCs), while synaptic GABA regulates NPC development into mature granule neurons [140]. Thus, it is possible to postulate that the use of drugs that modulate the level of non-synaptic GABA could represent a strategy to regulate neuroblast production and migration.

#### 3.1.5. Glutamate

It has been shown that glutamate is a regulator of adult neurogenesis [141]. In particular, a role for metabotropic mGlu5 receptors (mGluR5) and NMDA receptors in promoting neurogenesis has been documented [114]. In vitro, cultured NPCs express functional mGluR3 and mGluR5 [142], and in vivo, mGlu5 receptors are expressed in embryonal and post-natal zones of active neurogenesis [143]. Adult mice lacking mGluR5, or treated with mGluR5 or mGluR3 antagonists, showed a dramatic reduction in the number of dividing neuro-progenitors in the SVZ and in the DG [142]. In line with these data, pharmacological activation of mGluR5s enhanced cell proliferation [142].

As regards NMDA receptors, it has been shown that in cultured NPCs, isolated from adult murine hippocampus, the activation of functional heteromeric NMDA receptors plays a crucial role in commitment to and differentiation of neurons [144]. In addition, NMDA receptor antagonists induce a long-lasting increase in the number of proliferating cells [145,146]. More recently, it has been reported that different level of NMDA receptor activation can promote proliferation or differentiation of hippocampal NPC [147]. Moreover, it has been shown that the deletion of NR2B-NMDA subunit receptor from adult-born neurons in DG impairs a neurogenesis-dependent form of long-term potentiation (LTP). In detail, NR2B deletion did not affect cell survival, but reduced dendritic complexity [148]. In line with the role of mGlu5 and NMDA receptors in adult neurogenesis, + it has been recently shown that TLQP-62 (VGF C-terminal peptide) treatment induces generation, but not differentiation, of early progenitor cells in the DG, with a mechanism that requires the activity of mGluR5 and NMDA receptors [149].

All these data demonstrate that glutamate can influence proliferation and neuronal commitment, and acts as a positive regulator of neurogenesis.

**Table 2:** Neurotransmitters: beyond the neuronal functions.

Neurotransmitters	Receptors	Effects	References
**dopamine**	n.d.	Increases NPC proliferation in SVZ	[115,116,117]
	D2-like	Promotes entry in S phase	[120]
	D2-like	Decreases NPC proliferation	[118,119]
	D1-like	Inhibits the entry in S phase	[121]
	D3	Inhibits the maturation of OPC	[122]
**serotonin**	n.d.	Depletion decreases proliferation in DG and SVZ	[123]
	5-HT1A	Blockade of receptors decreases proliferation in DG	[124]
	n.d.	Increases NPC proliferation in DG	[125,126]
	n.d.	Blocks OPC maturation	[127]
	5-HT	Increases levels of trophic factors	[128,129,130,131]
**norepinephrine**	n.d.	Depletion decreases NPC proliferation but not differentiation in DG	[132]
	β3-AR	Increases proliferation of NPC in DG	[133]
	a2-AR/β-AR	Balance in receptor activity regulates NPC activity in DG	[134]
	n.d.	Reduces NPC proliferation in SVZ	[135]
**gaba**	extra synaptic GABA-A	Inhibits NPC proliferation in SVZ	[136]
	extra synaptic GABA-A	Regulates NPC production and migration	[137,138,139]
	extra synaptic GABA-A	Maintains NPC quiescence in DG	[140]
	synaptic GABA-A	Promotes NPC maturation in DG	[140]
**glutamate**	n.d.	Regulates adult neurogenesis	[114,142]
	mGluR5	Promotes Neurogenesis in DG	[142,149]
	NMDA	Promotes commitment and diferentiation in DG	[145,146,147,149]
	NR2B	Increases dendritic arborization and contributes LTP induced neurogenesis	[148]

### 3.2. Neurotransmitters Influence Glioma

Neurotransmitters play functions alternative to synaptic transmission in physiological conditions as well as in pathological conditions, such as brain cancer.

The most common brain tumor in adults is the glioblastoma (GBM) [1], a high-grade glioma with high proliferation and invasion rate. Within the tumor mass, stem cells with protein expression profile similar to healthy neural stem cells have been identified [150]. Thus, similar to NPC, glioma cells express the receptors and respond to neurotransmitters, such as monoamines, GABA and glutamate (see Table 3).

#### 3.2.1. Monoamines

The expression of genes related with monoamine pathway is modulated in GBM specimens (TCGA and REMBRANDT database), and it reveals a significant correlation with patient survival [151]. Recently, it has also been shown that gene polymorphisms in monoamine oxidase A (MAO-A), the enzyme responsible for monoamine degradation, are associated with GBM in a case-control study performed in males [152]. This association is of interest, also considering that GBM in males has a higher incidence, with a male:female ratio of 1.6:1 [153]. Furthermore, in a preclinical study it was demonstrated that MAO-A inhibitors decreased the progression of temozolomide-resistant glioma, increasing the survival time in mice, and decreasing tumor cell viability and invasion capabilities in vitro [154].

#### 3.2.2. Dopamine

In GBM, the dopamine receptor D2 (DRD2) is overexpressed in comparison with other dopamine receptors and the overexpression is associated with a poor prognosis [155]. In addition, DRD2 activation is responsible for the mitogenic signaling in GBM through the sequential activation of RAP1-GTP, Raf-1 release, and MEK/ERK signaling [156].A selective inhibitor of DRD2 (ONC201), now in clinical trial for different solid tumors and recurrent GBM (NCT02525692) [157], inhibits the cellular proliferation of cancer stem cell-enriched neurospheres obtained from primary and recurrent GBM, and induces apoptosis in stem cell-like glioma cells [158,159].

In a recent study, it was demonstrated that the activation of DRD2 together with epidermal growth factor receptor (EGFR) contributes to spheroid formation and maintenance [160]. In line with these results, DRD2 silencing with specific shRNA reduced U87MG cell growth [155]. These studies show a tumorigenic effect linked to the activation of D2R in GBM, that increases the rate of cell proliferation. However, it has also been demonstrated that the administration of dopamine precursor (levodopa) inhibits cell growth and induces vascular normalization through the reprogramming of M2-polarized macrophages into M1/anti-tumoral phenotype, in a rat glioma model [161]. Accordingly, treatment of primary microglial cultures with a DRD2/DRD3 agonist, increases the release of nitrite and IFN-γ [162]. Dopamine receptors are also expressed on dendritic cell, on natural killer (NK) cells, macrophages, microglia, intermediate monocytes, neutrophils, and eosinophils [163]. All these immune cells participate to both cognitive functions and the modulation of glioma microenvironment thus, these data highlight additional putative immunomodulatory roles of dopamine signaling [164,165,166].

#### 3.2.3. Serotonin

The data available on the role of serotonin in GBM onset and progression show a complex relationship. Emerging evidence demonstrate that the selective serotonin reuptake inhibitors (SSRI) have anti-tumoral effects in animal models [167,168,169,170], implying a beneficial effect of higher parenchymal serotonin concentration in GBM. However, a recent retrospective study conducted on 497 GBM patients, treated with SSRI to inhibit serotonin transport by SERT, failed to find an association between the use of SSRI and increased overall survival [171].

It has been reported that serotonin also exhibits growth stimulatory effect on several types of human tumor cell lines, including glioma [172,173]. In glioma, the positive effect of serotonin on cell growth appears mediated through 5-HT1 and 5-HT2 receptors more than other serotonin receptors [173]. For example, the activation of 5-HT2A receptors with selected agonists increased the propagation and migration of C6 rat glioma cells [174]. In addition, physiological concentration of serotonin enhanced both mRNA and protein levels of GFAP in rat C6 glioma cells, indicating that serotonin might induce the differentiation of these cells [175]. Moreover, serotonin, through the activation of 5-HT2 receptors, increases the release of glial cell line-derived neurotrophic factor (GDNF) in by C6 cells [176]. It has also been shown that GDNF promotes the survival, activation, proliferation, and migration of several glioma cell lines, suggesting that GDNF release could be important for glioma formation. In line with these results, it has been suggested that multiple autocrine signals were produced by secreted factors, among which a possible serotonin-stimulated GDNF loop could contribute to the invasive properties of the most aggressive forms of glioma [177].

#### 3.2.4. Norepinephrine

Scant and contradictory information is available on the role of norepinephrine in the onset and/or progression of glioma. Both murine and human GBM express adrenergic receptors [178,179]. A recent study demonstrated that U251 cells increase proliferation upon β-adrenergic receptor activation, via the ERK1/2 pathway and matrix metalloproteinase (MMP)-2 and MMP-9 expression [180]. Conversely, the activation of β2-adrenergic receptor inhibits the proliferation of 1321N1 astrocytoma cells [181]. More recently, it was shown that norepinephrine inhibits the migration and invasion of human GBM cells through the decrease in MMP-11 expression and activity [182]. However, in a retrospective study on a cohort of 218 GBM patients, a correlation was not found between the use of beta-blockers and the overall survival or progression-free survival [183]. More studies are needed to better understand the impact of norepinephrine on tumor cell proliferation and migration, also in light of the effect of stress in tumor progression in many types of cancers [184].

#### 3.2.5. GABA

The expression of GABA-A subunit correlates with the malignancy grade of the tumor [185]; however, the functional expression of the GABAR is restricted to low-grade gliomas and not detected in GBM. In vitro and in vivo studies in low-grade glioma, demonstrated that GABAA-R activation induces chloride efflux and cell depolarization, resulting in reduced rate of cell proliferation, possibly sustaining cell quiescence in specific tumor cell populations responsible for recurrence and therapy resistance [186].

The interstitial level of GABA appears crucial to attenuate glioma growth in vivo: an analysis with ultra-sensitive 3-tesla magnetic resonance demonstrated that GABA levels are decreased in IDH1-mutated low-grade glioma foci compared to the contralateral hemisphere of patients [187].

In line with this result, a comprehensive study on GBM databases showed that expression of three GABA related genes—glutamate decarboxylase 1 (GAD1) and 2 (GAD2) and 4-aminobutyrate aminotransferase (ABAT)—are lower in mesenchymal GBM, indicating that a decreased production and possibly an increased catabolism may be linked to poor outcome [188]. Thus, on the basis of these emerging evidence, more data from pre-clinical studies are necessary to elucidate the activity of GABA signaling in glioma growth.

#### 3.2.6. Glutamate

GBM patients showed increased levels of brain glutamate ranging from 100 to 600 μM [189]. It has been shown that these levels are responsible for seizure and excitotoxity of peritumoral cells in glioma bearing mice models [190].

GBM cell lines and primary cultures from patients express high levels of the class II metabotropic glutamate receptor (mGluR2/3) [191]. However, it has been shown that low expression of mGluR3 correlates with a better survival rate in GBM [192], and that the blockade of mGluR2/3 activity reduces U87MG growth halting cell cycle progression [193] and reduces the proliferation of primary cultures of GBM [194].

GBM cells express low level of the Ca^2+^ impermeable GluR2 AMPAR [195]. It was reported that AMPAR activation increases high grade glioma proliferation and migration through the Ca^2+^-dependent activation of Akt/PKB signaling pathway; these effects were inhibited by the use of NBQX, a selective AMPAR inhibitor [195]. AMPAR activation promotes perivascular invasion via β1 integrin–dependent adhesion to the extracellular matrix in vitro and in vivo systems [196], and the activation of NMDA receptor affects MMP-2 activity and proliferation of U251MG and U87MG cells [197]. Recent evidence demonstrated that neuronal activity affects glioma cell functions. Neuronal activity promotes glioma proliferation through the release of the soluble factor neuroligin-3 (NLGN3) [198,199], and peritumoral neurons directly interact with glioma cells through AMPA-mediated synapses that drive tumor growth and invasion [200,201], opening new stimulating perspectives to the understanding of the complex glioma-brain parenchyma interactions.

**Table 3:** Neurotransmitters influence glioma.

Neurotransmitters	Receptors	Effects	References
**dopamine**	D2	Regulates survival and cell proliferation	[155,156]
	D2	Increases cancer stem cell-enriched spheroid proliferation	[158,159,160]
**serotonin**	5-HT1, 5-HT2	Increases cell proliferation	[172,173]
	5-HT2A	Increases cell proliferation and migration	[174]
	n.d.	Induces cell differentiation	[175]
	5-HT2A	Increases the release of neurotrophic factor GDNF	[176]
**norepinephrine**	β	Increases proliferation	[180]
	β2	Inhibits proliferation	[181]
	n.d.	Inhibits migration and invasion	[182]
**gaba**	GABA-A	Reduces cell proliferation sustaining cell quiescence	[186]
**glutamate**	mGluR2/3	Regulates cell growth	[193,194]
	GluR1/GluR4	Increase cell proliferation and migration	[195]
	AMPA	Promotes perivascular invasion	[196]
	NMDA	Increases proliferation	[197]
	n.d.	Drives tumor growth and invasion	[198,199,200,201]

## 4. Do Gut Microbiota-Derived Neurotransmitters Affect Glioma Development?

**Figure 2:**
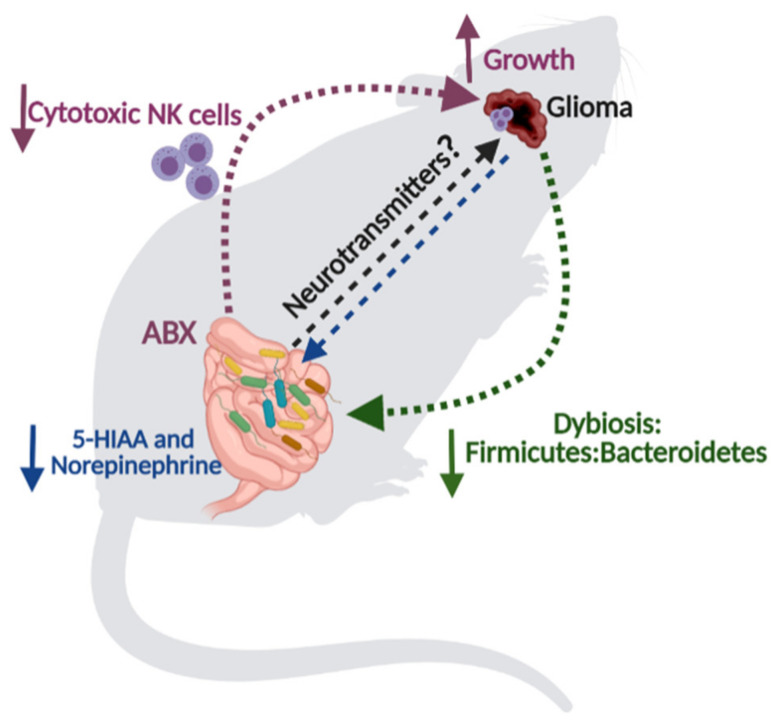
Gut microbiota-glioma crosstalk. Oral antibiotics (ABX)-treated mice show a reduced frequency and cytotoxicity of Natural Killer (NK) cells at systemic and brain tumor level, and an increased glioma growth (purple dotted arrow). Glioma-bearing mice show gut microbiota dysbiosis with a reduced ratio of Firmicutes and Bateroidetes (green dotted arrow), and a decreased fecal level of norepinephrine and serotonin metabolite, 5-hydroxyindoleacetic acid (5-HIAA) (blue dotted arrow). Perspective for gliomas: could brain neurotransmitter levels, modulated by gut microbiota, represent a key determinant for tumor progression? (black dotted arrow).

Despite the physiological variations of microbiota composition reported during life in healthy individuals, the gut microbiota is relatively constant. However, gut dysbiosis, a dramatic change in the balance of bacterial ecosystem, could lead to over-representation of some specific bacterial populations which can favor chronic inflammation and immunosuppression [202]. Recently, it has been described that gut microbiota can influence acute CNS diseases, such as stroke [31], or neurodegenerative disorders such as Parkinson’s disease [27], MS [29,30], and Alzheimer’s disease [28]. However, the relationship between the gut-brain axis and glioma development has been only recently investigated [38,203,204] (Figure 2). It has been shown that chronic antibiotics treatment increases glioma growth, with a reduction of the cytotoxic NK cell subsets both in the tumor microenvironment and peripheral organs [38]. It has been also reported that microbiota elimination impacts microglial phenotype [71], with a shift toward a more immune-suppressive, pro-tumoral state [38]. More recently, another study demonstrated that glioma induces alterations in the microbiota reducing the *Firmicutes:Bacteroidetes* ratio, increasing the relative abundance of phylum *Verrucomicrobia*, with a resulting increase in the genus *Akkermansia* and its more common species *Akkermansia muciniphila* [203]. In addition, in gliomabearing mice altered levels of fecal SCFAs and neurotransmitters have been shown. In particular, reduced levels of 5-HIIA and norepinephrine were found in the fecal samples by targeted metabolomic analysis [204]. All together, these studies demonstrate that the bidirectional axis between the intestine and the brain is also a determinant of glioma biology.

## 5. Conclusions

Considering (i) the ability of gut microbiota to modify neurotransmitter levels in the brain (mediators); (ii) the influence of neurotransmitters on cell proliferation in physiological condition (NPC cells) and tumor glioma cells, we propose that the ability of gut microbiota to modulate neurotransmitter levels could represent a key determinant in brain tumor progression (Figure 2).

Additional research is needed in order to examine and determine if the crosstalk between microbiota and glioma mediated by neurotransmitters, could have a clinical significance. For future research, we propose to investigate how the modulation of specific bacterial species (see Table 1) might result in selective neurotransmitter alteration in the brain, with possible effects on gliomagenesis. Further, manipulation of microbiota composition by means of dietary habits, environmental stimuli, the use of pre-and pro-biotics, and fecal transplantation might represent a strategy to modify neurotransmitters levels in the brain, hampering glioma growth.

## Figures and Tables

**Table 1:** Neurotransmitters levels modulated by gut microbiota.

Neurotransmitters	Microbes	Effects	References
**dopamine**	*Enterococcus faecium* and *Lactobacillus rhamnosus*	Increased levels in healthy mouse brain	[77]
	n.d.	Increased turnover rate in the brain striatum of GF mice	[12]
	*Lactobacillus plantarum*	Increased levels in the brain striatum of GF mice	[79]
	*Enterococcus faecalis*, *Enterococcus faecium*	Increased levels in brain, blood, and feces of the pseudo GF mice	[78]
	*Enterococcus faecalis*, *Enterococcus faecium*, *Proteus mirabilis*, *Lactobacillus acidophilus*	Increase level in vitro	[78]
	*Clostridium* species	Restored levels in the lumen of GF mice	[73]
**serotonin**	*Lactobacillus plantarum*	Increased levels in the brain striatum of GF mice	[79]
	n.d.	Increased turnover rate in the brain striatum of GF mice	[12]
	n.d.	Increased levels in hippocampus of male GF mice	[80]
**norepinephrine**	*Clostridium* species	Restore levels in the lumen of GF mice	[73]
	n.d	Increased turnover rate in the brain striatum of GF mice	[12]
**gaba**	n.d.	Reduced levels in gut lumen of GF mice	[81]
	*Lactobacillus rhamnosus*	Modulated GABA receptor mRNA expression in healthy mice brains	[70]
	*Enterococcus faecium* and *Lactobacillus rhamnosus*	Increased levels in healthy mice brains	[77]
	*Lactobacillus rhamnosus*	Increased brain levels in healthy mice	[82]
**glutamate**	*Lactobacillus rhamnosus*	Increased brain levels in healthy mice	[82,83]

## Abbreviations

GBM	Glioblastoma
GF	Germ Free
GABA	Gamma-aminobutyric acid
CNS	Central nervous system
5-HT	5-hydroxytryptamine
GI	Gastrointestinal
SCFAs	Short-chain fatty acids
BDNF	Brain derived neurotrophic factor
ASD	Autism spectrum disorder
PD	Parkinson’s disease
AD	Alzheimer’s disease
MS	Multiple sclerosis
MIA	Maternal immune activation
ASO	α-synuclein overexpressing
Aβ	Amiloid–β
EAE	Experimental autoimmune encephalomyelitis
MCAO	Middle cerebral artery occlusion
EECs	Enteroendocrine cells
ANS	Autonomic nervous system
ENS	Enteric nervous system
LPS	Lipopolysaccharide
GLP	Glucagon like peptide
LBP	LPS binding protein
BBB	Blood-brain barrier
MCTs	Monocarboxylate transporters
HPA	Hypothalamic-pituitary-adrenal
CCK	Cholecystokinin
NO	Nitric oxide
BH4	Tetrahydrobiopterin
TH	Tyrosine hydroxylase
5-HIAA	5-hydroxyindoleacetic acid
Kyn	Kynurine
NMDA	N-methyl-D-aspartate
AMPA	Alpha-amino-3-hydroxy-5-methyl-4-isoxazole propionic acid
SZ	Subventricular zone
OPC	Oligodendrocyte progenitor cells
DG	Dentate Gyrus
β3AR	β3 adrenergic receptor
GFAP	Glial fibrillary acidic protein
NPCs	Neuronal precursor cells
mGluR5	metabotropic glutamate 5 receptor
LTP	Long-term potentiation
MAO-A	Monoamine oxidase A
DRD-2	Dopamine receptor D2
EGFR	Epidermal growth factor receptor
NK	Natural killer
SSRI	Selective serotonin reuptake inhibitor
GDNF	Glial cell line-derived neurotrophic factor
MMP	Matrix metalloproteinase
GAD	Glutamate decarboxylase
ABAT	4-aminobutyrate aminotransferase
NLGN3	Neuroligin-3
